# Squalenyl Hydrogen Sulfate Nanoparticles for Simultaneous Delivery of Tobramycin and an Alkylquinolone Quorum Sensing Inhibitor Enable the Eradication of *P. aeruginosa* Biofilm Infections

**DOI:** 10.1002/anie.202001407

**Published:** 2020-05-11

**Authors:** Duy‐Khiet Ho, Xabier Murgia, Chiara De Rossi, Rebekka Christmann, Antonio G. Hüfner de Mello Martins, Marcus Koch, Anastasia Andreas, Jennifer Herrmann, Rolf Müller, Martin Empting, Rolf W. Hartmann, Didier Desmaele, Brigitta Loretz, Patrick Couvreur, Claus‐Michael Lehr

**Affiliations:** ^1^ Helmholtz Institute for Pharmaceutical Research Saarland Helmholtz Center for Infection Research 66123 Saarbrücken Germany; ^2^ INM—Leibniz Institute for New Materials 66123 Saarbrücken Germany; ^3^ Faculté de Pharmacie Institut Galien Paris Sud Université Paris-Saclay, UMR CNRS 8612 92296 Châtenay-Malabry France; ^4^ German Centre for Infection Research (DZIF) Partner Site Hannover-Braunschweig 66123 Saarbrücken Germany; ^5^ Department of Pharmacy Saarland University 66123 Saarbrücken Germany; ^6^ Current address: Department of Bioengineering School of Medicine University of Washington Seattle WA 98195 USA; ^7^ Current address: Kusudama Therapeutics Parque Científico y Tecnológico de Gipuzkoa 20014 Donostia-San Sebastián Spain

**Keywords:** biofilms, drug delivery, *Pseudomonas aeruginosa*, quorum sensing inhibitors, tobramycin

## Abstract

Elimination of pulmonary *Pseudomonas aeruginosa* (PA) infections is challenging to accomplish with antibiotic therapies, mainly due to resistance mechanisms. Quorum sensing inhibitors (QSIs) interfering with biofilm formation can thus complement antibiotics. For simultaneous and improved delivery of both active agents to the infection sites, self‐assembling nanoparticles of a newly synthesized squalenyl hydrogen sulfate (SqNPs) were prepared. These nanocarriers allowed for remarkably high loading capacities of hydrophilic antibiotic tobramycin (Tob) and a novel lipophilic QSI at 30 % and circa 10 %, respectively. The drug‐loaded SqNPs showed improved biofilm penetration and enhanced efficacy in relevant biological barriers (mucin/human tracheal mucus, biofilm), leading to complete eradication of PA biofilms at circa 16‐fold lower Tob concentration than Tob alone. This study offers a viable therapy optimization and invigorates the research and development of QSIs for clinical use.

## Graphical abstract

**Breaking through**: Tob and QSI (1) co‐loaded squalenyl hydrogen sulfate nanoparticles (SqNPs) are shown to act on all stages of *P. aeruginosa* infections to eradicate bacterial infection [(a–c) and (e)] and penetrate biofilm, preventing its formation and recurrence (d).

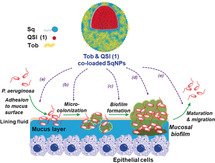

*Pseudomonas aeruginosa* (PA) is one of the most virulent pathogens causing nosocomial infections worldwide.^1^ Especially, this biofilm forming bacterium is the predominant life‐threatening pathogen in cystic fibrosis (CF) patients, leading to high morbidity and mortality.^2^ Inhaled antibiotics reduce the frequency of exacerbations, significantly decrease bacterial density in airway secretions, recover pulmonary function, and most importantly improve the quality of life of patients with PA‐derived pulmonary infections. Despite intensive focus on the discovery of new anti‐infectives and pulmonary delivery strategies, the currently available inhalation therapeutics cannot entirely eradicate bacterial lung infections.^3^ Therefore, the development of new and improved treatment strategies in the context of severe pulmonary infections remains an unmet medical need. To address this, inhalation therapeutics will have to overcome the challenges of pulmonary drug delivery, mainly including poor water‐solubility, poor absorption, and fast clearance, while providing sustained release of anti‐infectives at a concentration higher than the minimum inhibiting concentration both in the lungs and inside the biofilm infection.^3^, ^4^ PA is also able to form biofilms composed of attached bacterial cells covered within a matrix of extracellular polymeric secretions.^5^ Moreover, it could further shift to a mucoid phenotype in CF patients in later stages of infection, which forms overexpressed alginate biofilms.^5^ These are strong penetration‐limiting biological barriers protecting bacteria from anti‐infective agents and leading to antimicrobial resistance development.^5^, ^6^

Depending on the bacterial density, PA strains may produce complex biofilm infections in the host by upregulating the expression of several virulence factors through inter‐bacterial chemical communications, so called quorum sensing (QS) systems.^7^ The PA‐specific transcriptional multi‐virulence factor regulator (pqsR) is one of the QS systems in PA, which coordinates the production of pyocyanin, elastase B, hydrogen cyanide, and biofilm formation.^7^ Pyocyanin generates reactive oxygen species, which benefit the bacterial colonization.^8^ Moreover, the PqsR‐controlled QS metabolites prompt PA to convert to a metabolically less active state at which they are less or even not sensitive to antibiotics.^7^, ^8^ QS systems are thus considered as attractive targets in combatting PA infections.^9^, ^10^ A potent pqsR inverse agonistic QSI (Scheme 1, QSI (1)) was discovered and synthesized for such purpose.^9^, ^10^ QSI (1) strongly interferes with PA virulence without affecting cell growth, and thus has been proposed as a complement to antibiotics.^10^, ^11^ The simultaneous delivery of antibiotic and QSI is hypothesized to improve biofilm eradication.^12^, ^13^, ^14^ Nevertheless, the efficacy of QSI (1) is restricted by its poor water‐solubility and the concomitant limitations in terms of pulmonary delivery. Furthermore, the chemical synthesis of excipients and formulation methods of a few reported delivery systems for anti‐infective‐combination remains cumbersome and poorly scalable.^3^

**Scheme 1:**
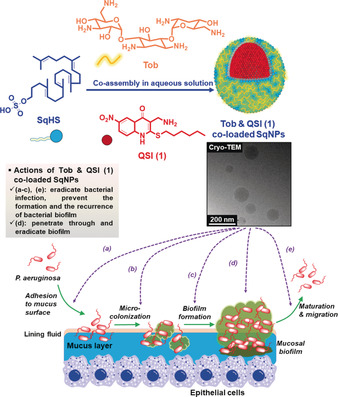
Schematic illustration of the co‐assembly of Tob and QSI (1) co‐loaded SqNPs, their ultrastructure by Cryo‐TEM image, and their proposed actions at all stages of PA respiratory infections.

To overcome the aforementioned limitations, we describe herein a novel nano‐carrier system based on a newly synthesized amphiphilic lipid, squalenyl hydrogen sulfate (SqHS) (Scheme 1). SqHS nanoparticles (SqNPs) are easily formed in aqueous media due to SqHS's unique nano‐assembling property, which allows the simultaneous delivery of both the polycationic antibiotic tobramycin (Tob, the widely used first‐line therapy in the treatment of PA associated infections) and a hydrophobic QSI (1). This approach enables the co‐delivery of both anti‐infectives and therefore is expected to provide high drug availability at and inside the infection sites, thereby providing enhanced antimicrobial activity at all stages of the infection (Scheme 1).

To the best of our knowledge, the anti‐infectives co‐loaded SqNPs described display a remarkably higher drug loading capacity compared to previously reported drug delivery systems having similar a size range.^3^ Particularly, the drug co‐loaded SqNPs of 200 nm allowed the maximum Tob and QSI (1) loading capacity at 30 % and circa 10 %, respectively. Detailed SqNPs characteristics are summarized in Figure S3 in the Supporting Information, while the morphology of the Tob and QSI (1) co‐loaded SqNPs could be observed by Cryo‐TEM (Scheme 1).

We firstly investigated the efficacy of QSI (1)‐loaded SqNPs on PA strain PA14 wild type (wt). Figure 1 A shows an improvement by a factor of four in pyocyanin inhibitory efficacy when using QSI (1)‐loaded SqNPs compared to free QSI (1), whereas the control drug‐free SqNPs did not display any activity (Figure S6). This is a result of the better availability of hydrophobic drug in an aqueous environment, which encourages the use of SqNPs for the delivery of QSI (1).

**Figure 1:**
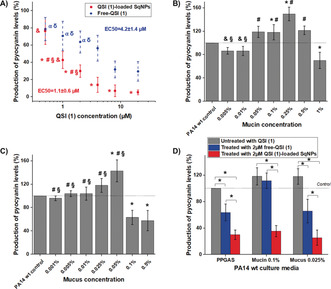
Production of pyocyanin levels compared to control PA14 wild type (wt): A) of samples treated with free QSI (1) in blue (^α^, ^δ^p<0.001 vs. samples treated with 20 and 8 μm QSI (1)), and with QSI (1)‐loaded SqNPs in red (*, ^#^, ^§^, ^&^ *p*<0.001 vs. samples treated with equivalent 0.5, 20, 8 and 4 μm QSI (1)); B) in the presence of mucin (*, ^#^, ^&^, ^§^ *p*<0.001 vs. controls, mucin concentration 1, 0.25 and 0.1 %); C) in the presence of human mucus (*, ^#^, ^§^ *p*<0.001 vs. controls, human mucus concentration 0.5 and 0.1 %). D) Comparison of pyocyanin production levels of PA14 wt grown in PPGAS medium (proteose peptone glucose ammonium salt), in the presence of mucin 0.1 % and mucus 0.025 %, treated with 2 μm QSI (1) as either free form or loaded in SqNPs (*p<0.001). All control samples were grown in PPGAS medium, which was also used as diluent for mucin and human mucus. All data are presented as mean±SD, at least three independent experiments performed in triplicate each.

In the context of PA‐induced pulmonary infections, airway mucus is always the first contact with the host at the early stages of infection. Mucus is a protecting layer that entraps and clears foreign materials, including bacteria, out of the lungs.^15^, ^16^ Unfortunately, mucus clearance is often compromised in disease states such as CF, which might promote the development of bacterial resistance.^17^ Hence, we investigated the pyocyanin production of PA14 wt in the presence of mucin, the main organic component of mucus, and in the presence of crude, native human tracheal mucus. The results are shown in Figure 1 B,C. Interestingly, our study pointed out that, at the highest tested concentrations of both mucin and mucus, the pyocyanin production was inhibited, while bacteria growth was not affected in both conditions. Although the tested mucin/mucus concentrations are lower than the reported ones in healthy airway mucus and CF sputum,^18^, ^19^ the observed phenomenon corroborates the biochemical effects of mucus, which inhibits cell‐to‐cell communication and, as recently reported by Wheeler et al., also influences the biofilm formation.^17^ On the contrary, when decreasing mucin or mucus concentrations, which corresponds to an increase of the ratio between bacteria population to mucin or mucus amount (i.e., the setting of infection), the pyocyanin production was not inhibited, yet significantly promoted. Notably, the pyocyanin levels were enhanced by a factor of 1.5 when incubating PA14 wt with mucin or mucus at 0.25 % and 0.05 % concentration, respectively. This might be, in turn, of relevance to the immunosuppression of PA once its population increases in the host. Furthermore, the presence of mucin or mucus might limit the water‐solubility and permeation of the hydrophobic QSI. Hence, we compared the pyocyanin inhibitory effect of 2 μm QSI (1) either in its free form or loaded in SqNPs on PA14 wt, which was grown in PPGAS medium, or medium either supplemented with 0.1 %*w*/*w* mucin or 0.025 %*w*/*w* mucus concentrations. These chosen concentrations were lower than the most pyocyanin‐promoting conditions to minimize the effects on water‐solubility of QSI (1). Figure 1 D shows that the inhibition efficacy of free QSI (1) is notably abolished in the presence of 0.1 % mucin. On the contrary, QSI (1)‐loaded SqNPs still showed a significant inhibitory effect, which is comparable to those observed in the assays performed in PPGAS medium. At lower mucus concentrations, the activity of the free form QSI (1) was not impaired. As mucin or mucus at the studied concentrations would not have significant effect on the viscosity, the observations in mucin‐containing medium could be due to the other effects. For instance, the glycoprotein mucin has hydrophobic regions in its structure,^16^ which could adsorb QSI (1) molecules and limit their contact with the bacteria. The QSI (1)‐loaded SqNPs are, by contrast, well‐dispersed and show consistently better inhibitory efficacy in all tested media.

Despite the attractive potency of QSIs in limiting bacterial virulence and increasing antimicrobial susceptibility, clinical studies are relatively scarce and no such drug product has yet reached market approval.^20^ The lack of adequate in vitro and in vivo models with suitable endpoints might be a possible reason for this. Moreover, QSIs as monotherapy could be limited to prophylaxis, which is of less relevance compared to the medical needs of clinically confirmed infections.^21^ Encouraged by the benefits of QSI (1)‐loaded SqNPs, we investigated the biofilm eradicating efficacy by simultaneously delivering QSI (1) and Tob. As shown in Figure 2 A, PA14 wt biofilms could only be completely eradicated at Tob concentrations of 200 μg mL^−1^ or higher, which is dramatically higher than the efficacious concentration against planktonic bacteria (3.125–6.25 μg mL^−1^). This limited efficacy might be due to strong interaction with the biofilm matrix, which results in slow and incomplete Tob permeation into the biofilm core.^22^ Furthermore, bacteria might become metabolically less active in the biofilm thereby being less sensitive to Tob. Together with the fast clearance of Tob from the lungs after inhalation, these appear as the main reasons limiting eradication of PA‐associated chronic lung infections. Tob‐loaded SqNPs neutralized the positive charge of Tob and showed a slight improvement in efficacy with a determined MBEC value at 200 μg mL^−1^ (Figure 2 B). The free form mixture of Tob and QSI (1), in which QSI (1) concentration was kept constantly at 20 μm, performed better in combatting PA14 wt biofilm compared to the treatments of Tob mentioned above (Figure 2 C). In addition to a decrease of the Tob MBEC value to 100 μg mL^−1^, the overall bacteria viability (cfu mL^−1^) in all samples treated with lower Tob concentrations was reduced compared to those treated in the absence of QSI (1). These results represent an encouraging proof of concept that Tob and QSI (1) in combination act synergistically. Hence, we hypothesized that simultaneous co‐delivery of Tob and QSI (1) by the same SqNPs, would further enhance the PA‐eradicating efficacy by facilitated biofilm crossing and thus better bioavailability of both drugs inside the biofilm. Therefore, we treated PA biofilms with Tob and QSI (1) co‐loaded into SqNPs in which the QSI (1) concentration was kept constant at 20 μm. Remarkably, the biofilm‐eradicating Tob concentration was even further reduced from 200 μg mL^−1^ (free Tob) to 12.5 μg mL^−1^, which is nearly 16‐fold lower (Figure 2 D). Notably, no biofilm eradicating effect at all was observed after administering either drug‐free SqNPs, 20 μm QSI (1) free form or loaded in SqNPs (Figure S7). Moreover, PA biofilms treated with the drugs co‐loaded SqNPs at Tob concentrations below 12.5 μg mL^−1^ had significantly lower average cfu mL^−1^ values than that of samples treated with the same Tob concentrations in other regimens. These results prove the significant advantage of the co‐delivery of Tob and QSI (1) as enabled by such nanotechnology. Besides enhanced biofilm penetration, it may be speculated that the nano‐carrier enhances the solubility of QSI (1) and provides a sustained release of both actives by maximizing the antibiotic effect of Tob and enabling PA biofilm eradication at significantly lower antibiotic concentrations.

**Figure 2:**
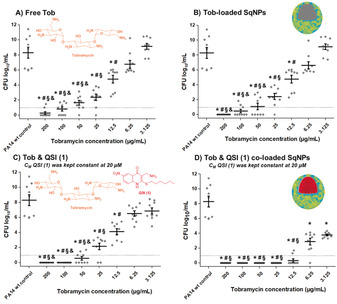
Minimum biofilm eradicating concentration (MBEC) assay on PA14 wt biofilm grown in PPGAS medium for 24 h: biofilms were treated with A) free Tobramycin (Tob), B) Tob‐loaded SqNPs, C) free Tob and free QSI (1), and D) Tob and QSI (1) co‐loaded SqNPs. The concentration of QSI (1) was 20 μm and kept constant in all assays using QSI (1). After a 24 h treatment, efficacy was assessed by determination of colony‐forming units per millilitre (cfu mL^−1^). Cfu mL^−1^ values are depicted logarithmically for *n*=3 experiments with technical triplicates each. Untreated PA14wt biofilm, PA14 wt biofilms treated with either drug‐free SqNPs, or free QSI (1), or QSI (1)‐loaded SqNPs were served as controls. The dotted lines indicate the detection limit. (*, ^#^, ^§^, ^&^ *p*<0.001 vs. controls, samples treated with Tob 3.125, 6.25, 12.5 μg mL^−1^).

We investigated the permeation of QSI (1) either as free form or loaded in SqNPs through 24 h‐old PA14 wt biofilms grown on transwell membrane (Figure 3 A). The limited amount of QSI (1) permeating through the membrane over time, from the apical to the basolateral compartment (Figure 3 B), clearly demonstrates the barrier function of PA biofilms. Incomplete permeation of QSI (1) was observed using either form. Interestingly, the permeation of QSI (1)‐loaded SqNPs was 5‐fold higher than that of free QSI (1) after 8 h incubation. We visualized the transport of SqNPs through a PA14 wt biofilm using live CLSM. The presence of SqNPs at the bottom layer of biofilm after 4–8 h incubation (Figure 3 C and Videos S1–S3) demonstrate the penetration of this delivery system through biofilm. Moreover, SqNPs were found well distributed deep inside biofilm after 8 h incubation (Figure 3 D) suggesting improved availability of loaded drugs in the biofilm core.

**Figure 3:**
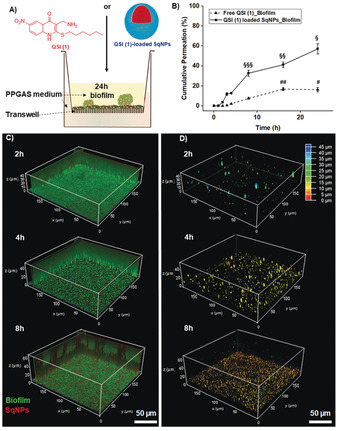
A) Schematic illustration of the transport study of free QSI (1) or QSI (1)‐loaded SqNPs through a 24 h‐old PA14 wt biofilm grown in PPGAS medium. B) Cumulative permeation (%) of QSI (1) collected in the basolateral compartment normalized to the permeation of free QSI (1) through the bare transwell membrane, pore size 400 nm (^#^, ^##^ *p*<0.001 vs. concentrations measured at 0–8 h; and ^§^„ ^§^ *p*<0.001 vs. concentrations measured at 0–8 h, 0–4 h, and 0–3 h). 3D‐projections of biofilm penetrating Tob‐loaded SqNPs (diameter 190 nm, Tob concentration 12.5 μg mL^−1^), SqNPs were labelled with Nile‐red: C) the transport of red‐labelled SqNP through biofilms of PA modified with GFP (green fluorescent protein) after 2, 4, and 8 h incubation (see also Videos S1–S3); D) the topographic distribution of SqNPs after 2, 4, and 8 h incubation is shown through a colour code.

Relevant chronic PA‐mediated lung infection animal models are challenging to establish. Moreover, currently described animal models have been reported for not providing adequate endpoints and not properly reflecting the biological environment of biofilm infections.^21^, ^23^ Our in vitro data, however, clearly demonstrates the synergistic effects of simultaneously delivering Tob and QSI (1) through SqNPs, thus provides an adequate endpoint and hopefully invigorates the research and development of QSI for clinical use.

In summary, we have described a novel nano‐carrier system allowing simultaneous encapsulation and delivery of both Tob and QSI at high loading capacities. We have addressed the effects of mucin and mucus, respectively, on PA's pyocyanin production. We have also demonstrated the biofilm penetration of SqNPs and the superior antimicrobial activity of drug‐loaded SqNPs compared to free anti‐infectives. Consequently, co‐delivery of Tob and QSI (1) by SqNPs showed a remarkable synergistic effect, enabling complete eradication of PA biofilm at circa 16‐fold lower concentration of Tob compared to this drug alone. The SqHS synthesis and SqNPs preparation is facile and scalable. Furthermore, SqNPs were found to be well compatible with human cells (IC_50_ % in MTT assay >300 ug mL^−1^; Figure S8), and also showed no toxicity on zebrafish embryos at the highest tested concentration (200 ug mL^−1^, Table S3). Hence, we seek to advance this approach toward clinical translation of new therapeutic modalities based on optimized QSI candidates and their co‐delivery with suitable antibiotics.

## Conflict of interest

The authors declare no conflict of interest.

## Supporting information

As a service to our authors and readers, this journal provides supporting information supplied by the authors. Such materials are peer reviewed and may be re‐organized for online delivery, but are not copy‐edited or typeset. Technical support issues arising from supporting information (other than missing files) should be addressed to the authors.

Supplementary

Supplementary

Supplementary

Supplementary

## References

[1] E. Yusuf , B. Van Herendael , W. Verbrugghe , M. Ieven , E. Goovaerts , K. Bergs , K. Wouters , P. G. Jorens , H. Goossens , Ann. Intensive Care 2017, 7, 72. 28664350 10.1186/s13613-017-0296-z PMC5491427

[2] M. J. Richards , J. R. Edwards , D. H. Culver , R. P. Gaynes , Infect. Control Hosp. Epidemiol. 2000, 21, 510–515. 10968716 10.1086/501795

[3] D.-K. Ho , B. L. B. Nichols , K. J. Edgar , X. Murgia , B. Loretz , C.-M. Lehr , Eur. J. Pharm. Biopharm. 2019, 144, 110–124. 31493510 10.1016/j.ejpb.2019.09.002

[4] D. Brown , Nat. Rev. Drug Discovery 2015, 14, 821–832. 26493767 10.1038/nrd4675

[5] H. C. Flemming , J. Wingender , Nat. Rev. Microbiol. 2010, 8, 623–633. 20676145 10.1038/nrmicro2415

[6] J. W. Costerton , P. S. Stewart , E. P. Greenberg , Science 1999, 284, 1318–1322. 10334980 10.1126/science.284.5418.1318

[7] M. A. Welsh , H. E. Blackwell , FEMS Microbiol. Rev. 2016, 40, 774–794. 27268906 10.1093/femsre/fuw009 PMC5007280

[8] E. C. Pesci , B. H. Iglewski , A. Latifi , M. Foglino , A. Lazdunski , Trends Microbiol. 1997, 5, 132–134. 9141185 10.1016/S0966-842X(97)01008-1

[9] C. Lu , C. K. Maurer , B. Kirsch , A. Steinbach , R. W. Hartmann , Angew. Chem. Int. Ed. 2014, 53, 1109–1112; 10.1002/anie.201307547 24338917

[10] A. A. M. Kamal , L. Petrera , J. Eberhard , R. W. Hartmann , Org. Biomol. Chem. 2017, 15, 4620–4630. 28513746 10.1039/c7ob00263g

[11] G. Brackman , P. Cos , L. Maes , H. J. Nelis , T. Coenye , Antimicrob. Agents Chemother. 2011, 55, 2655–2661. 21422204 10.1128/AAC.00045-11 PMC3101409

[12] V. Roy , M. T. Meyer , J. A. I. Smith , S. Gamby , H. O. Sintim , R. Ghodssi , W. E. Bentley , Appl. Microbiol. Biotechnol. 2013, 97, 2627–2638. 23053069 10.1007/s00253-012-4404-6

[13] H. M. H. N. Bandara , M. J. Herpin , D. Kolacny , A. Harb , D. Romanovicz , H. D. C. Smyth , Mol. Pharm. 2016, 13, 2760–2770. 27383205 10.1021/acs.molpharmaceut.6b00360

[14] M. Hema , S. A. Princy , V. Sridharan , P. Vinoth , P. Balamurugan , M. N. Sumana , RSC Adv. 2016, 6, 45938–45946.

[15] M. Yang , S. K. Lai , Y. Y. Wang , W. Zhong , C. Happe , M. Zhang , J. Fu , J. Hanes , Angew. Chem. Int. Ed. 2011, 50, 2597–2600; 10.1002/anie.201006849 PMC3100893 21370345

[16] X. Murgia , B. Loretz , O. Hartwig , M. Hittinger , C.-M. Lehr , Adv. Drug Delivery Rev. 2018, 124, 82–97. 10.1016/j.addr.2017.10.009 29106910

[17] K. M. Wheeler , G. Cárcamo-Oyarce , B. S. Turner , S. Dellos-Nolan , J. Y. Co , S. Lehoux , R. D. Cummings , D. J. Wozniak , K. Ribbeck , Nat. Microbiol. 2019, 4, 2146–2154. 31611643 10.1038/s41564-019-0581-8 PMC7157942

[18] B. Button , H. P. Goodell , E. Atieh , Y. C. Chen , R. Williams , S. Shenoy , E. Lackey , N. T. Shenkute , L. H. Cai , R. G. Dennis , et al., Proc. Natl. Acad. Sci. USA 2018, 115, 12501–12506. 30420506 10.1073/pnas.1811787115 PMC6298066

[19] A. G. Henderson , C. Ehre , B. Button , L. H. Abdullah , L. H. Cai , M. W. Leigh , G. C. DeMaria , H. Matsui , S. H. Donaldson , C. W. Davis , et al., J. Clin. Invest. 2014, 124, 3047–3060. 24892808 10.1172/JCI73469 PMC4072023

[20] A. Thomann , A. G. G. De Mello Martins , C. Brengel , M. Empting , R. W. Hartmann , ACS Chem. Biol. 2016, 11, 1279–1286. 26882081 10.1021/acschembio.6b00117

[21] K. Lewis , Nat. Rev. Drug Discovery 2013, 12, 371–387. 23629505 10.1038/nrd3975

[22] B. Cao , L. Christophersen , M. Kolpen , P. Ø. Jensen , K. Sneppen , N. Høiby , C. Moser , T. Sams , PLoS One 2016, 11, 0153616. 10.1371/journal.pone.0153616 PMC4839563 27100887

[23] D. M. Cornforth , J. L. Dees , C. B. Ibberson , H. K. Huse , I. H. Mathiesen , K. Kirketerp-Møller , R. D. Wolcott , K. P. Rumbaugh , T. Bjarnsholt , M. Whiteley , Proc. Natl. Acad. Sci. USA 2018, 115, E5125–E5134. 29760087 10.1073/pnas.1717525115 PMC5984494

