# Supplementary material for: Squalenyl Hydrogen Sulfate Nanoparticles for Simultaneous Delivery of Tobramycin and an Alkylquinolone Quorum Sensing Inhibitor Enable the Eradication of P. aeruginosa Biofilm Infections
Source: Angew Chem Int Ed Engl. 2020 May 11;59(26):10292–6. doi: 10.1002/anie.202001407 (PMC7317969; doi:10.1002/anie.202001407)
Supplement: Supplementary file 1 — Supplementary [file ANIE-59-10292-s001.pdf]

Angewandte Chemie
Eine Zeitschrift der Gesellschaft Deutscher Chemiker

Supporting Information

Squalenyl Hydrogen Sulfate Nanoparticles for Simultaneous Delivery of Tobramycin and an Alkylquinolone Quorum Sensing Inhibitor Enable the Eradication of *P. aeruginosa* Biofilm Infections

*Duy-Khiet Ho, Xabier Murgia, Chiara De Rossi, Rebekka Christmann, Antonio G. Hüfner de Mello Martins, Marcus Koch, Anastasia Andreas, Jennifer Herrmann, Rolf Müller, Martin Empting, Rolf W. Hartmann, Didier Desmaele, Brigitta Loretz, Patrick Couvreur, and Claus-Michael Lehr**

anie_202001407_sm_miscellaneous_information.pdf
anie_202001407_sm_Video_S1.mp4
anie_202001407_sm_Video_S2.mp4
anie_202001407_sm_Video_S3.mp4

Table of Contents

A. Materials 2

B. Methods and Supporting Results 3

1. Synthesis of squalenyl hydrogen sulfate (SqHS) 3

2. Nanoparticles preparation and characterization. 5

2.1. Preparation of free-drug SqNPs 5

2.2. Preparation of drug-loaded SqNPs 5

2.3. Isothermal titration calorimetry 6

2.4. NPs characterization 7

2.5. Cryo-TEM 7

3. Quantification of drug loading 7

4. Release study 9

5. MICs (minimum inhibitory concentrations) determination 10

6. Pyocyanin assay 10

7. MBECs (minimum biofilm eradicating concentrations) determination 12

8. QSI (1) diffusion studies 14

9. Laser scanning confocal microscopy 14

10. Cell viability: MTT assay 15

11. Zebrafish embryo toxicity assay 16

12. Statistical analysis 17

A. Materials

All chemicals were purchased from Sigma-Aldrich unless otherwise specified. Yeast extract was obtained from Fluka. Bacto™ Tryptone were obtained from BD Biosciences. Luria Bertani (LB) agar was obtained from Carl Roth. Gibco® PBS was obtained from Life Technologies. Purified water was prepared by a Milli-Q water purification system (Merk Millipore, Billerica, MA).

All bacteria strains were from ATCC or DSMZ, the German Collection of Microorganism and Cell Cultures GmbH (PA14 = DSMZ19882; PA GFP = ATCC15692GFP) and cultured in minimal proteose peptone glucose ammonium salt (PPGAS) medium. PPGAS was composed of 1 g/L NH_4Cl, 1.5 g/L KCl, 19 g/L Tris-HCl, 10 g/L peptone, 5 g/L glucose and 0.1 g/L $MgSO_4$•$7H_2O$. The medium was adjusted to pH 7.2 ± 0.2 and sterilized before use. Agar solution was composed of 15.5 g/L LB agar, 10 g/L peptone, 5 g/L NaCl and 5 g/L yeast extract, the solution was sterilized before being plated.

The cell line A549 (ATCC, CCL-185), passage number between 27 and 56, was used in this work. The cell line was grown and maintained in T75 tissue culture flasks, with RPMI 1640 medium supplemented with 10% (v/v) of inactivated FBS (Fetal Bovine Serum). The cells were fed every other day and trypsinized once reached about 80% confluence.

Zebrafish embryos of the AB wild-type line were below 120h post fertilization (hpf). The assay was not classified as an animal experiment according to EU Directive 2010/63/EU. Protocols for husbandry and care of adult animals are in accordance with the German Animal Welfare Act (§11 Abs. 1 TierSchG).

B. Methods and Supporting Results

1. Synthesis of squalenyl hydrogen sulfate (SqHS)

Squalene hydrogen sulfate was synthesized from 1,1',2-trisnorsqualenol as described in Scheme S1. 1,1',2-trisnorsqualenol was synthesized from squalene via 1,1′,2-trisnorsqualenic aldehyde according to reported methods.[1–3] 1,1',2-trisnorsqualenol (1 molar equivalent) was solubilized in DMF at 10% w/w concentration. TEA.SO_3 (sulfur trioxide triethylamine complex) (1.1 molar equivalent) was slowly dropped into the solution at room temperature, under N_2 condition. Following 16h reaction at 60 °C, an appropriate amount of MilliQ water was slowly added for 1h to quench the system. DMF and water were then removed under reduced pressure. The crude product solution was subsequently diluted in MeOH, and NaOH 1N was added under stirring. MeOH was then removed under reduced pressure, and an appropriate amount of MilliQ water was added. The pure SqSH was then extracted using an excess amount of ethyl acetate. The solution was then dried over $MgSO_4$, and solvents were removed completely under reduced pressure at 40 °C. The isolated yield was 95%. The pure SqSH was analyzed by ^{1}H-NMR (Figure S1), ^{13}C-NMR (Figure S2) and MS (mass spectroscopy) before further use. ^{1}H-NMR ($CDCl_3$, 300 MHz, Figure S2), δ ppm: 5.15-5.09 (m, 5H, C*H*=C(CH_3)), 4.13-4.08 (m, 2H, *C*H_2OSO_3H), 2.09-1.94 (m, 18H, =C(CH_3)C*H*$_2$C*H*$_2$), 1.80-1.75 (m, 2H, *C*H_2CH_2O), 1.69 (s, 3H, =CH(C*H*$_3$)$_2$), 1.64 (s, 15H, =C(C*H*$_3$)CH_2);^{13}C-NMR ($CDCl_3$, 300 MHz, Figure S3) δ ppm: 135.31 (C, CH=*C*(CH_3)CH_2), 135.21 (C, CH=*C*(CH_3)CH_2), 135.00 (C, CH=*C*(CH_3)CH_2), 134.14 (C, CH=*C*(CH_3)CH_2), 131.33 (C, CH=*C*(CH_3)CH_2), 124.91 (CH, *C*H=C(CH_3)), 124.58 (CH, *C*H=C(CH_3)), 124.44 (2CH, *C*H=C(CH_3)), 124.29 (CH, *C*H=C(CH_3)), 69.93 (CH_2, *C*H_2OSO_3H), 39.98 (CH_2, =C(CH_3)*C*H_2*C*H_2), 39.92 (CH_2, =C(CH_3)*C*H_2*C*H_2), 39.88 (CH_2, =C(CH_3)*C*H_2*C*H_2), 35.73 (CH_2, =C(CH_3)*C*H_2*C*H_2), 28.54 (2CH_2, =C(CH_3)*C*H_2*C*H_2), 27.95 (CH_2, =C(CH_3)*C*H_2*C*H_2), 27.27 (CH_2, =C(CH_3)*C*H_2*C*H_2), 26.93 (CH_2, =C(CH_3)*C*H_2*C*H_2), 26.87 (CH_2, =C(CH_3)*C*H_2*C*H_2), 25.83 (CH_3, CH=C(*C*H_3)$_2$),), 17.81

(CH_3, $=C(CH_3)CH_2$), 16.25 (CH_3, $=C(CH_3)CH_2$), 16.16 (CH_3, $=C(CH_3)CH_2$), 16.13 (2 CH_3, $=C(CH_3)CH_2$); MS (ESI-) *m/z*(%) 465.3 (100) [M - H]$^-$.

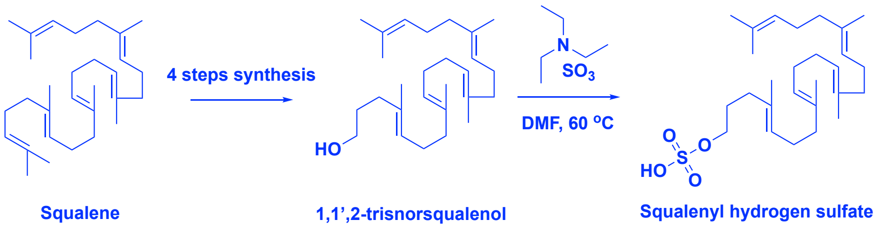

Scheme S1. Synthetic scheme of squalenyl hydrogen sulfate (SqHS)

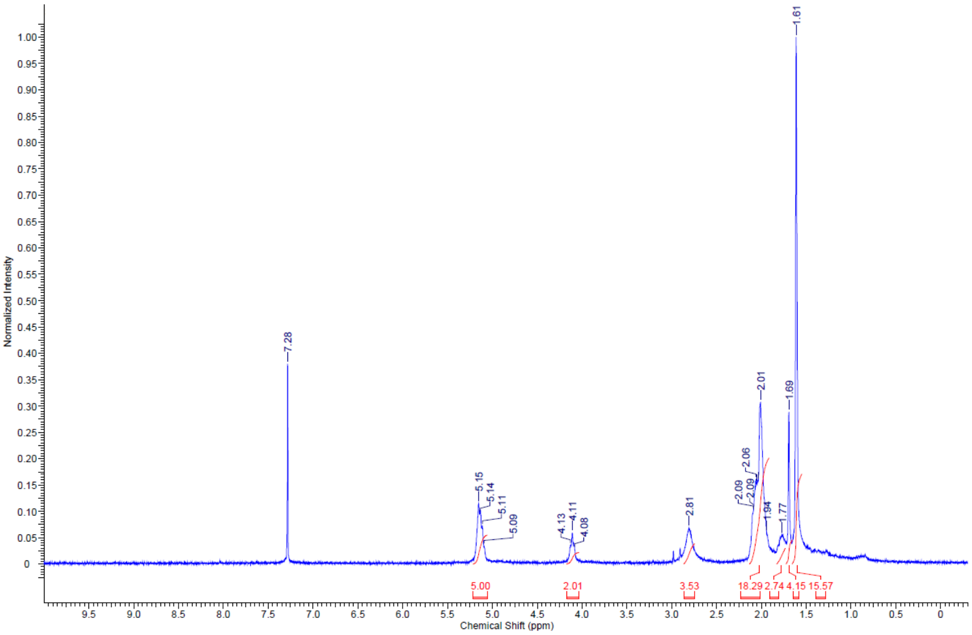

Figure S1. ^{1}H NMR spectrum of SqHS

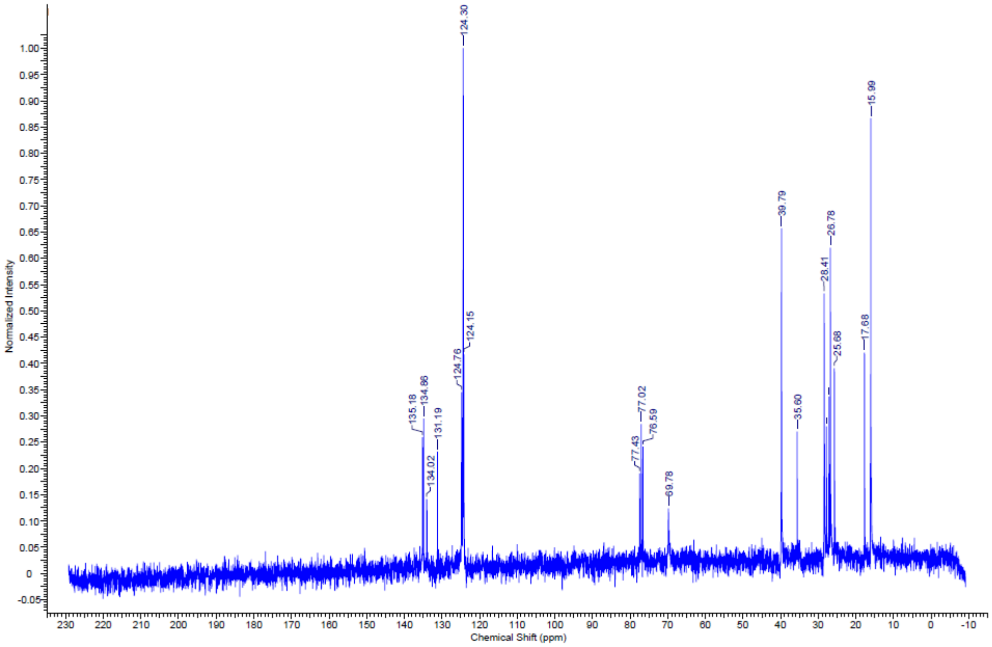

Figure S2. ^{13}C NMR spectrum of SqHS

2. Nanoparticles preparation and characterization.

Free-drug and drug-loaded squalenyl hydrogen sulfate nanoparticles (SqNPs) were prepared using a single step nanoprecipitation method.

2.1. Preparation of free-drug SqNPs

SqHS was solubilized in THF (tetrahydrofuran) (10 mg/mL), which was then dropped slowly into 1 mL of MilliQ water under magnetic stirring (THF/water 1:10 v/v). After organic solvent evaporation, a colloidal dispersion of SqNPs in aqueous medium was obtained. Final concentration of SqNPs solution could be tuned by changing the initial concentration of SqHS in THF.

2.2. Preparation of drug-loaded SqNPs

Hydrophobic *quorum* sensing inhibitor QSI (1)-loaded SqNPs were prepared using the same procedure, in which QSI (1) was solubilized in the SqHS solution in THF. The optimal QSI (1)/SqHS ratio was 1:9 (w/w), which allowed the maximum

encapsulation efficiency (EE%) and loading capacity (LC%) of QSI (1) in SqNPs. Hydrophilic tobramycin (Tob) was, in turn, solubilized in aqueous solution which was subsequently loaded on SqNPs surface via charge interaction during the precipitating procedure. The optimal Tob/SqHS ratio 3:7 (w/w) allowed the maximum EE%, LC%, and long-term colloidal stability.

Tob and QSI (1) co-loaded SqNPs were prepared by simultaneously co-assembling SqHS with Tob and QSI (1) using the above procedure. The Tob concentration loaded in SqNPs (ranging from 3.125 – 200 µg/mL) used in the latter biofilm assay was varied by changing the initial concentration.

2.3. Isothermal titration calorimetry

Interaction between SqNPs and the cationic Tob was investigated by isothermal titration calorimetry (ITC) using a NanoITC 2G (TA Instruments, Eschborn, Germany), aiming to prove the exposure of hydrogen sulfate groups on NPs surface and to optimize the EE% and LC% of Tob in SqNPs. Briefly, Tob and drug-free SqNPs were prepared in Milli-Q water and degassed prior to experiments. A solution of Tob (0.5 mg/mL) was prepared in a 250 µL syringe and used to saturate 1.5 mL of SqNPs at a concentration of 0.0625 mg/mL filled in the sample cell. Following an initial delay of 300 sec, the Tob solution was repeatedly injected into the sample cell, 14.86 µL each injection, with a spacing of 1000 sec between injections, and at a reference power of 10 µCal/s. The thermogram and thermodynamic parameters were generated by subtracting the heat of dilution of Tob (250 µL of the solution 0.5 mg/mL in 1.5 mL milliQ water), followed by fitting using the One Set of Sites model in the data analysis software NanoAnalyze. The free energy of binding (ΔG) was calculated using the equation $\Delta G = \Delta H - T\Delta S$,

where ΔH is the enthalpy change, T is temperature (Kelvin), and ΔS is the change in entropy. All measurements were performed at 25 °C.

2.4. NPs characterization

The characterization (hydrodynamic size, PDI, and ζ-potential) of the nanoparticles was measured by Dynamic Light Scattering (DLS, Zetasizer Nano-ZS, Malvern Instruments, Worcestershire, U.K.) equipped with a 4 mW He−Ne laser employing a wavelength of 633 nm and a backscattering angle of 173° at 25 °C. The reported size represents the z-average hydrodynamic diameter (intensity based) of three measurements.

2.5. Cryo-TEM

Morphology of nanoparticles was observed by cryo-TEM. Briefly, 3 µL of the sample solution was placed on a holey carbon grid (S147-4, Plano, Wetzlar, Germany), plotted for 2 seconds, and plunged into -165 °C liquid ethane. The frozen sample was then transferred under liquid nitrogen to a Gatan (Pleasonton, CA, United States) model 914 cryo-TEM sample holder and investigated using a JEOL (Akishima, Tokio, Japan) JEM-2100 LaB6 TEM at T=-170°C under low dose conditions. Bright field images were acquired using a Gatan Orius SC1000 CCD camera.

3. Quantification of drug loading

The loaded quantity of Tob and QSI (1) in SqNPs were determined indirectly by measuring of the unloaded molecules in the supernatant (loaded amount = initial amount – amount in the supernatant). Quantification of Tob and QSI (1) were carried out using liquid chromatography with tandem mass spectrometry (LC-MS/MS). The system was operated by the standard software Xcalibur. To quantify Tob, a reverse phase C18 Accucore RP-MS (150 x 2.1 mm) column (Thermo Scientific, Waltham, MA, USA) was used as stationary phase. . The column temperature was 30

°C. The mobile phase was composed of solvent A (water containing 0.1% v/v trifluoroacetic acid, TFA) and solvent B (acetonitrile containing 0.1% v/v TFA), solvent ratio A:B was 95:5, and the flow rate was 300 µL/min, controlled by an Accela 1250 Pump. A standard curve was run at Tob concentrations (2.5 – 80 µg/mL) in PBS. The quantification of QSI (1) was done using a method reported by Nafee *et al.*[4]

The EE% and LC% were calculated according to the following equations:

$$\text{EE\%} = \frac{Weight\ of\ encapsulated\ drug\ in\ NPs}{Initial\ weight\ of\ used\ drug}\ \text{x}\ 100 \qquad \text{(S1)}$$

$$\text{LC\%} = \frac{Weight\ of\ \ drug\ in\ NPs}{Weight\ of\ NPs}\ \text{x}\ 100 \qquad \text{(S2)}$$

"Weight of NPs" was calculated as: Weight of NPs = Weight of polymeric materials + Weight of encapsulated drug in NPs.

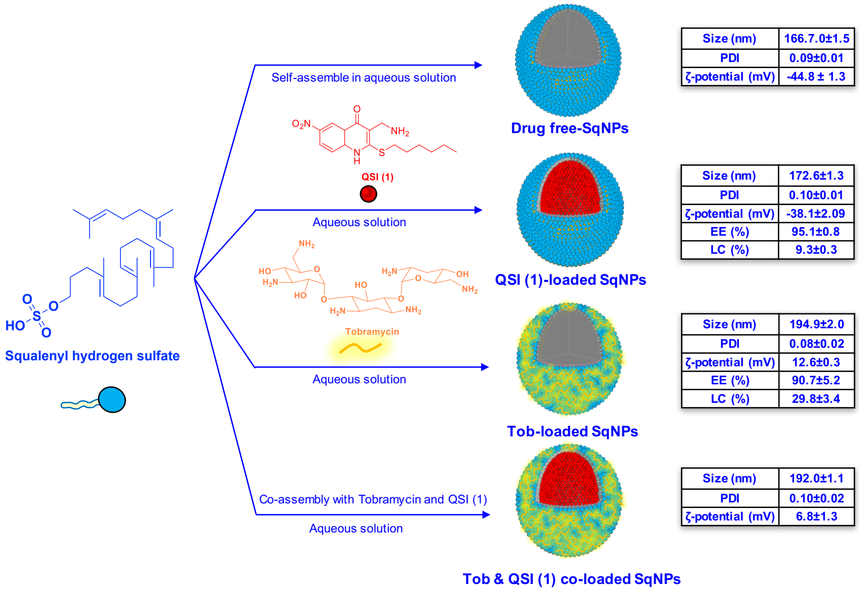

Figure S3. Drug-free and drug-loaded SqNPs formulations and their characteristics.

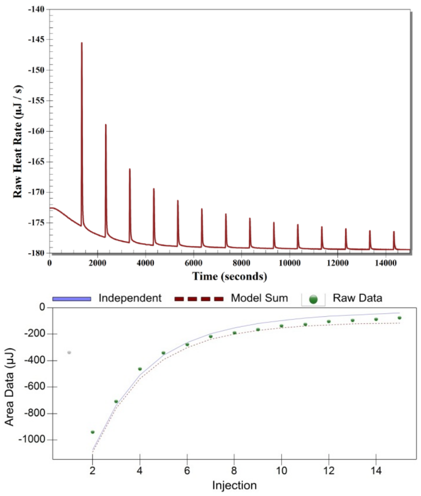

ΔH (kJ/mol)	-54,14 ± 0,45
ΔS (J/mol.K)	-106,2 ± 1,32
-TΔS (kJ/mol)	31,66 ± 0,50
ΔG (kJ/mol)	-22,47 ± 0,05
Kd (M)	1,155E-4

Figure S4. Isothermal titration calorimetry (ITC): titration of Tob (0.5 mg/mL) into SqNPs (0.0625 mg/mL).

4. Release study

The release profiles of Tob and QSI (1) from the drugs co-loaded SqNPs having maximum LC% (~30% Tob and ~10% QSI(1)) were performed in PBS (pH 7.4) at 37 °C. Briefly, Tob and QSI (1) co-loaded SqNPs were diluted in PBS to obtain a final NPs concentration of 10% (w/w) and placed on a shaker at 400 rpm at 37 °C. The concentration of the released compounds was analyzed by timely collecting samples from the supernatant over 24 h. QSI (1) was extracted from the supernatant using ethyl acetate. The volume was kept constant by refilling with an identical volume of PBS. The cumulative released drug (%) over 24 h was determined. Three independent experiments were conducted in triplicates, and results expressed as the mean ± SD.

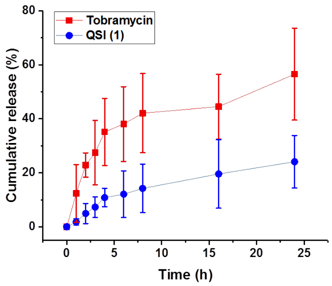

Figure S5. Cumulative release profiles of Tob and QSI (1) from the drugs co-loaded SqNPs

5. MICs (minimum inhibitory concentrations) determination

The antimicrobial properties of drug-free SqNPs, free QSI (1) prepared in dimethyl sulfoxide (DMSO), free Tob, QSI (1)-loaded SqNPs, Tob-loaded SqNPs, and Tob and QSI (1) co-loaded SqNPs were investigated by standard microbroth dilution assays with *Pseudomonas aeruginosa* (PA14 wild type strain) and *Pseudomonas aeruginosa* GFP (PA GFP, green fluorescent protein) in 96 well plates. A suspension of PA14 or PA GFP prepared from mid-log cultures in PPGAS medium was diluted to OD600 (absorption at 600 nm) 0.02, which corresponded to approximately 2×10^7 CFU/mL (CFU, colony-forming units). PBS served as a control. After incubation for 16 h at 37 °C, inhibitory concentration (IC) IC90 values were determined by sigmoidal curve fitting of absorption values (600 nm) that were measured on a Tecan microplate reader Infinite M200Pro (Tecan, Crailsheim, Germany). The IC90 values are defined as the compounds' concentrations at which the growth of bacteria is inhibited by 90%. Three independent experiments were conducted in triplicate.

6. Pyocyanin assay

Pyocyanin assay was performed as reported in previous works.[5,6] Briefly, PA14 culture was grown in PPGAS medium, overnight, at 37 °C, shaking at 200 rpm. The culture was then centrifuged at 7,450 *g*, washed twice with PBS and diluted to a final OD600 of 0.02 in fresh PPGAS, which corresponded to approximately 2×10^7 CFU/mL. The culture was distributed in 24 well-plate, 1.5 mL each well. Drug-free SqNPs, free QSI (1) prepared in DMSO, or QSI (1)-loaded SqNPs were added to bacteria-containing wells (1:100 dilutions). PA14 treated with PBS served as controls. After further incubation of 16 h under aerobic conditions, pyocyanin in 900 µL of bacteria culture was extracted as following procedure: (i) extraction with 900 µL of chloroform; (ii) collecting 800 µL of the chloroform solution; (iii) re-extracting with 250 µL of 0.2 M HCl to collect pyocyanin. Pyocyanin level was determined as the absorption at 520 nm of 80 µL of the extracted

aqueous solution, which was normalized to cell growth measured as OD600. 0.2 M HCl served as blank samples. Three independent experiments were conducted in triplicate. Drug-free SqNPs had no effect on PA14 pyocyanin production (Figure S6).

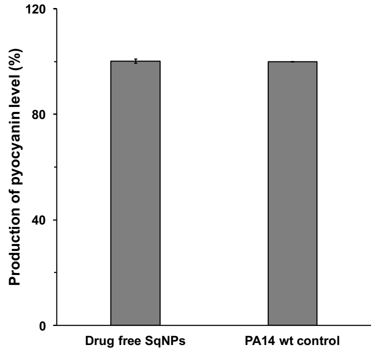

Figure S6. The production of pyocyanin levels compared to control PA14 wide type (wt) of the samples treated with 200 µg/mL drug free SqNPs.

Mucin or mucus was supplemented directly in PPGAS medium in the pyocyanin assay performed with these materials. Type II mucins from porcine stomach were purchased from Sigma. Undiluted human airway mucus was obtained using the tracheal tube method, as previously described.[7–9] Briefly, the tracheal tubes of several patients undergoing elective surgery with general anaesthesia were collected at the end of the procedure. Informed consent was obtained from all patients and the procedure was approved by the *Chamber of Medicine Doctor of the Saarland* (file number 19/15). Mucus was extracted from the tracheal tube by mild centrifugation (20 sec at 500g), freeze-dried and stored within sterile bags in a dry atmosphere.[10] The water content of undiluted mucus has been reported to be 95% and the mucin concentrations range between 8% and 22% of the solid content; mucus samples processed this way do not exhibit bacterial growth when incubated for 24 h in agar plates.[9] The extraction of known concentrations of pyocyanin in mucin and mucus solutions was performed to validate the assay procedure.

The growth of PA14 in PPGAS medium supplemented with mucin or mucus was also investigated prior to pyocyanin assay. In agreement with Müller and Murgia *et al.*[9] mucin and mucus did not inhibit PA14 growth at tested concentrations.

Table S1. Validation of pyocyanin extraction procedure from mucin/mucus supplemented PPGAS medium.

Initial pyocyanin in 900 µL mucin/mucus solution (µg)	Theoretical pyocyanin concentration after extraction process (µg/mL)	Actual pyocyanin concentration in HCl 0.2N after extraction process	
		from mucin 1% (w/w) solution (µg/mL) (*)	from mucus 0.5% (w/w) solution (µg/mL) (*)
3.5	12.5	9.72±0.39	9.77±0.35
7	25	26.68±0.73	26.58±0.79
14	50	53.29±1.20	53.71±0.63
28	100	108.83±0.74	107.89±0.64

(*) n=6

7. MBECs (minimum biofilm eradicating concentrations) determination

MBEC assay was performed according to a reported protocol with minor modification.[11] PA14 or PA GFP culture was grown in PPGAS medium, overnight at 37 °C shaking at 200 rpm. The culture was then centrifuged at 7,450 *g*, washed twice with PBS and diluted to a final OD600 of 3.0 in fresh PPGAS, which corresponded to approximately 10^{15} CFU/mL. The culture was distributed in 96 well-plate, 0.2 mL each well. The 96 well-plates were then kept under aerobic conditions (37 °C, without shaking) for 24h to allow biofilm formation. Afterwards, planktonic bacteria were removed, and the 24h-old biofilms were washed twice with PBS and fed with fresh PPGAS medium. Stock solution of drug free SqNPs, free QSI (1) prepared in DMSO, free Tob, QSI (1)-loaded SqNPs, Tob-loaded SqNPs, or Tob and QSI (1) co-loaded SqNPs was diluted (1:100 dilution) to bacterial biofilm-containing wells to obtain the defined final concentrations. Biofilm treated with PBS served as control. After further incubation for 24 h at 37 °C without shaking, all samples were washed twice with PBS, added 200 mL fresh PPGAS and sonicated to disperse the biofilm. Eradicating efficacy was assessed according to viable bacterial load

determined using dilution in PBS/0.05% Tween20 and plating on agar plates to count CFU after overnight incubation at 20 °C. Three independent experiments were conducted in triplicate.

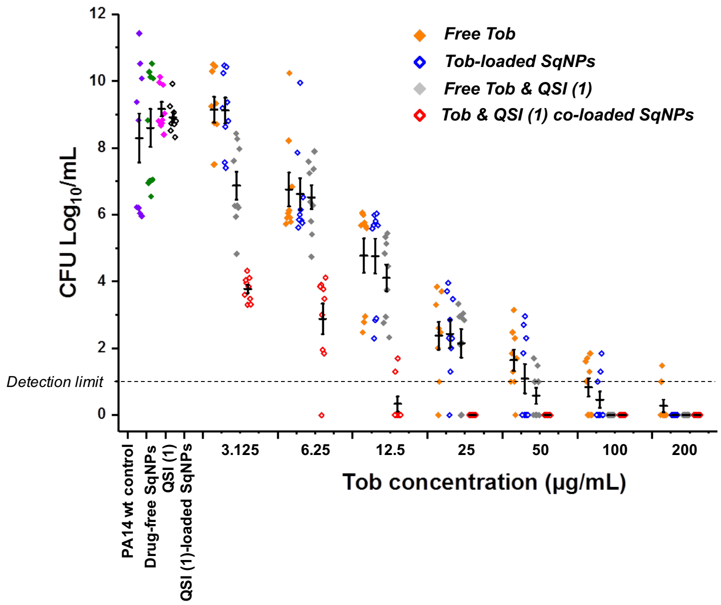

Figure S7. Minimum Biofilm Eradicating Concentration Assay.

PA14 wt biofilm grown in PPGAS for 24h were treated with (i) free Tob; (ii) Tob-loaded SqNPs; (iii) mixture of free Tob and free QSI (1); (iv) Tob and QSI (1) co-loaded SqNPs. Concentration of QSI (1) was kept constant at 20 µM in all experiments. Cfu/mL values are depicted logarithmically for n = 3 experiments with technical triplicates each. Untreated PA14 wt biofilm, and PA14 wt biofilm treated either with drug-free SqNPs, or with QSI (1), or QSI (1)-loaded SqNPs were served as controls. The dotted line indicates the detection limit.

Table S2. MIC90 values against planktonic PA14 and PA GFP; and MBEC values against PA GFP 24h-old biofilm.

Samples	MIC90 against PA14	MIC90 against PA GFP	MBEC against PA GFP 24h-old biofilm
Drug free SqNPs	> 200 µg/mL	> 200 µg/mL	> 200 µg/mL
QSI (1) prepared in DMSO	> 20 µM	> 20 µM	> 20 µM
QSI (1)-loaded SqNPs	> 20 µM [(a)]	> 20 µM [(a)]	> 20 µM [(a)]
Tob	3.125-6.25 µg/mL	3.125-6.25 µg/mL	> 200 µg/mL
Tob-loaded SqNPs	3.125-6.25 µg/mL [(b)]	3.125-6.25 µg/mL [(b)]	~ 200 µg/mL [(b)]
Tob and QSI (1) co-loaded SqNPs (QSI (1) concentration 20 µM)	3.125-6.25 µg/mL [(b)]	3.125-6.25 µg/mL [(b)]	n.a.

(a) QSI (1) concentration
(b) Tob concentration
n.a.: not applicable

8. QSI (1) diffusion studies

Experiments were performed using 96-well MultiScreen Permeability Filter Plates with a polycarbonate membrane (MPC4NTR10, Sigma, Germany) having a pore size of 0.4 µm. 24h-old PA14 biofilms were grown and washed as described above on the membrane prior to experiments. Inserts were placed into the companion plates and 20 µM of QSI (1) (equivalent to 7.1084 µg/mL) either free (prepared in DMSO) or loaded in SqNPs was then added to the apical compartment. The basolateral compartment was also filled with PPGAS. The solution from the basolateral compartment was collected at designated time points from 0 – 24h. Penetrate QSI (1) was isolated by extraction using ethyl acetate and dried overnight. QSI (1) was then dissolved in MeOH to determine its concentration using LC-MS/MS. The percentage of QSI (1) penetrating through biofilm was calculated based on the normalized 100% permeation of free QSI (1) prepared in DMSO through membrane. Three independent experiments were conducted in triplicate.

9. Laser scanning confocal microscopy

24h-old PA GFP biofilms were grown in PAA 96 well Imaging Plate FC, with fluorocarbon film bottom (PAA Laboratories, Pasching, Austria) as described above. For imaging, fluorescently-

labelled, Tob and Nile red co-loaded SqNPs (Tob concentration was 12.5 µg/mL) with a targeted size of ~200 nm were prepared and incubated with biofilms. The particles penetration and distribution in the 24h-old biofilm were investigated at 2h, 4h and 8h post-incubation. Imaging was performed using confocal laser scanning microscopy (CLSM, TCS SP 8, Leica, Mannheim, Germany) equipped with a 63x water immersion objective (HC APO CS2 63x/1.20). Z-stacks were performed with intervals of 0.5 µm using the plan-neofluar 40*x* / 1.3 oil DIC objective. Image analysis was performed using LAS X software (Leica Application Suite X; Leica, Mannheim, Germany). Videos of nanoparticle transport from the top to the bottom of the biofilm were generated using Image J (https://imagej.nih.gov/ij/).

Video S1. Tob and Nile red co-loaded SqNPs (Tob concentration was 12.5 µg/mL) transport in biofilm at 2h

Video S2. Tob and Nile red co-loaded SqNPs (Tob concentration was 12.5 µg/mL) transport in biofilm at 4h

Video S3. Tob and Nile red co-loaded SqNPs (Tob concentration was 12.5 µg/mL) transport in biofilm at 8h

10. Cell viability: MTT assay

A549 cells (10,000 cells) were grown for three days prior to the assay to allow approximately 80% cell confluency. Cells were further incubated with drug-free SqNPs pre-dispersed in HBSS (Hanks' Balanced Salt solution), at concentrations ranging from 50 – 1000 µg/mL, for 4 h, at 37 °C, and 5% CO_2. Followed by washing twice with PBS (Dulbecco's Phosphate Buffered Saline), MTT (Thiazolyl Blue Tetrazolium Blue) (0.5 mg/mL in HBSS) was incubated for 4 h in the dark. Subsequently, the supernatant was removed, and the formed formazan crystals were dissolved in DMSO. Finally, the absorbance was measured at 550 nm with a plate reader (Infinite M200Pro, Tecan, Germany). Cells incubated only with HBSS were used as negative control (corresponding to 100% cell viability) and cells treated with 1% TritonTM X-100 in HBSS medium were used as positive control (designated as 0% cell viability). The

percentage of viable cells was calculated in comparison to negative and positive controls. Three independent experiments were conducted in triplicate.

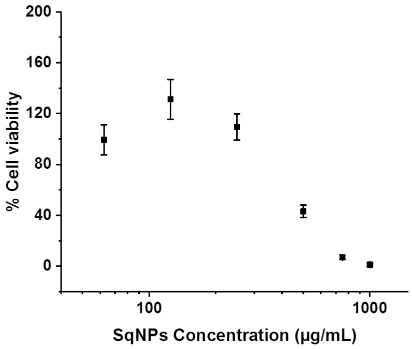

Figure S8. MTT assay: drug free SqNPs were incubated with A549 for 4 h. All measurements were conducted in triplicates. n=3, mean ± SD.

11. Zebrafish embryo toxicity assay

Three or four days post fertilization (dpf) embryos were collected and kept in a petri dish at 28 °C in 0.3*x* Danieau's medium (17 mm NaCl, 2 mm KCl, 1.8 mm $Ca(NO_3)_2$, 1.5 mm HEPES (pH 7.1 – 7.3), 0.12 mm $MgSO_4$ and 1.2 μm methylene blue) prior to experiments. The assay was performed in a 96-well plate with one embryo per well and 10 embryos per treatment group. Incubation solutions were prepared using 0.3*x* Danieau's medium. Single zebrafish embryos were placed in wells and directly incubated in the freshly prepared corresponding treatment solutions. The toxicity of tested formulation was determined by monitoring of developmental defects, heart rate, touch-evoked locomotion response and survival rate using microscopy.

Table S3. Zebrafish embryo toxicity assay

		Survival rate (%)	
Samples	Concentration	24h incubation	48h incubation
Drug free SqNPs (in 20% water)	100 µg/mL	100	90
Tob	200 µg/mL	100	100
QSI (1) (prepared in DMSO)	20 µM	100	100
DMSO control (1%)	-	100	100
Buffer control (20%)	-	100	100
Danieau's control	-	100	100

Developmental defects, abnormal heart rate and touch-evoked locomotion response were not observed in all tested conditions.

12. Statistical analysis

All values are given as mean ± standard error of the mean (SE), from at least three independent experiments which were conducted in triplicate. Statistical analysis was performed using OriginPro 2017 Software (OriginLab Corp., Massachusetts, USA). Significance was determined by One-way ANOVA as indicated in the respective figure captions.

Reference

[1] E. E. van Tamelen, T. J. Curphey, *Tetrahedron Lett.* **1962**, *3*, 121–124.

[2] C. Skarbek, L. L. Lesueur, H. Chapuis, A. Deroussent, C. Piochedurieu, A. Daville, J. Caron, M. Rivard, T. Martens, J. R. Bertrand, et al., *J. Med. Chem.* **2015**, *58*, 705–717.

[3] M. Ceruti, G. Balliano, F. Viola, L. Cattel, N. Gerst, F. Schuber, *Eur. J. Med. Chem.* **1987**, *22*, 199–208.

[4] N. Nafee, A. Husari, C. K. Maurer, C. Lu, C. De Rossi, A. Steinbach, R. W. Hartmann, C. M. Lehr, M. Schneider, *J. Control. Release* **2014**, *192*, 131–140.

[5] M. P. Storz, C. K. Maurer, C. Zimmer, N. Wagner, C. Brengel, J. C. De Jong, S. Lucas, M. Müsken, S. Häussler, A. Steinbach, et al., *J. Am. Chem. Soc.* **2012**, *134*, 16143–

16146.

[6] D. W. Essar, L. Eberly, A. Hadero, I. P. Crawford, *J. Bacteriol.* **1990**, *172*, 884–900.

[7] X. Murgia, H. Yasar, C. Carvalho-Wodarz, B. Loretz, S. Gordon, K. Schwarzkopf, U. Schaefer, C. M. Lehr, *Eur. J. Pharm. Biopharm.* **2017**, *118*, 79–88.

[8] D. K. Ho, S. Frisch, A. Biehl, E. Terriac, C. De Rossi, K. Schwarzkopf, F. Lautenschlaeger, B. Loretz, X. Murgia, C. M. Lehr, *Biomacromolecules* **2018**, *19*, 3499–3501.

[9] L. Müller, X. Murgia, L. Siebenbürger, C. Börger, K. Schwarzkopf, K. Sewald, S. Häussler, A. Braun, C. M. Lehr, M. Hittinger, et al., *J. Antimicrob. Chemother.* **2018**, *73*, 2762–2769.

[10] X. Murgia, H. Yasar, C. Carvalho-Wodarz, B. Loretz, S. Gordon, K. Schwarzkopf, U. Schaefer, C.-M. Lehr, *Eur. J. Pharm. Biopharm.* **2017**, *118*, 79–88.

[11] J. J. Harrison, C. A. Stremick, R. J. Turner, N. D. Allan, M. E. Olson, H. Ceri, *Nat. Protoc.* **2010**, *5*, 1236–1254.
